# A Conspicuous Nodule Over the Scalp: A Case Report

**DOI:** 10.7759/cureus.72141

**Published:** 2024-10-22

**Authors:** Ajay Dodeja, Hetal Karani, Sushil Pande, Apara Desai, Netra Mankar

**Affiliations:** 1 Department of Dermatology, Venereology and Leprology, N.K.P Salve Institute of Medical Sciences and Research Centre and Lata Mangeshkar Hospital, Nagpur, IND; 2 Department of Pathology, N.K.P Salve Institute of Medical Sciences and Research Centre and Lata Mangeshkar Hospital, Nagpur, IND

**Keywords:** appendageal tumor, hair follicle, histopathology, scalp, wide excisional biopsy

## Abstract

Trichilemmomas are benign and rare tumors of hair follicles that usually present as warty nodules and consist of clear and intermediate cells. A 34-year-old Indian man who initially presented with a complaint of a lesion over the left parietal region of the scalp and with a tendency to bleed on touch was clinically diagnosed with a pyogenic granuloma. It was managed by excising the lesion and the excised tissue was sent for histopathological examination, which was found to be a benign cutaneous adnexal neoplasm suggestive of a trichilemmoma. The patient is currently under follow-up and reports no recurrence at the end of six months.

## Introduction

Trichilemmomas were described as benign tumors from the outer sheath of the pilosebaceous follicle by Headington and French in 1962. They are further divided into two categories: intermediate cell type and clear cell type. Trichilemmomas usually appear on the face or neck and are characterized by asymptomatic, solitary, or numerous verrucous papules [1]. As they resemble other skin disorders, these lesions, while solitary, can frequently be clinically misdiagnosed [2]. A pyogenic granuloma (PG) is a benign vascular tumor that develops on the skin and mucous membranes. It is additionally referred to as lobular capillary hemangioma. PGs can develop spontaneously, at sites of injury, or within areas affected by capillary malformations [3]. We report a rare case of a trichilemmoma that clinically resembled that of a PG which was an unusual observation as per the published literature.

## Case presentation

A 34-year-old male patient presented to our outpatient department with a complaint of a painless bleeding nodular lesion over the scalp for a two-month duration. The patient gave a history of bleeding from the lesion while combing the hair. There was no history of hypertension, diabetes, bleeding disorders, or any significant medical illness. There was no history of trauma or surgery prior to the appearance of the lesion.

**Figure 1:**
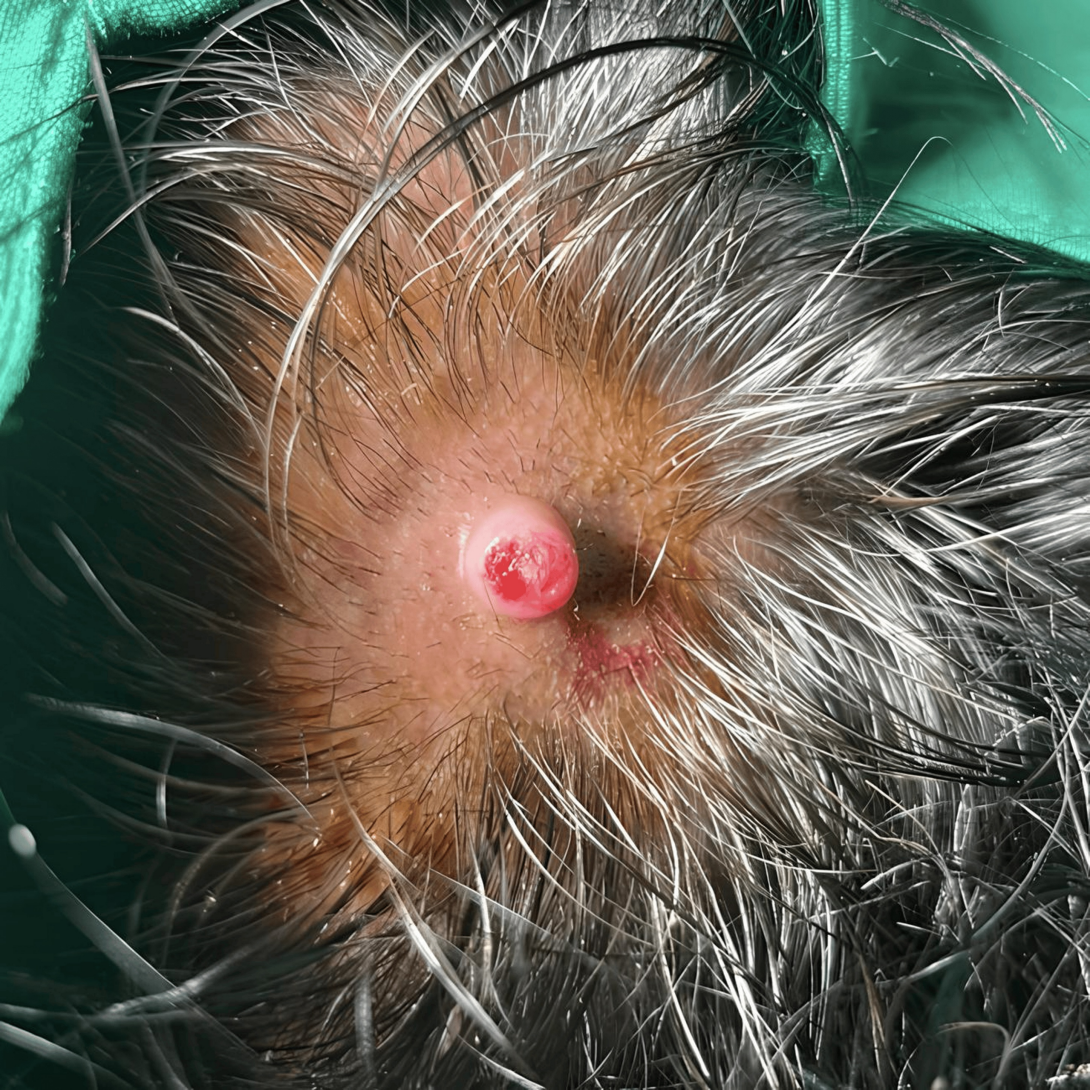
Single well-defined erythematous nodule with blood discharge and an erosive surface present over the left parietal region of the scalp.

General and systemic examination was normal. Cutaneous examination revealed a single non-tender erythematous nodule of approximate size of 1.5 cm x 0.8cm with central ulceration along with bloody discharge present over the left parietal region of the scalp (Figure 1). There was no regional lymphadenopathy. Based on clinical examination, a differential diagnosis of a PG or adnexal tumor was considered. A wide complete surgical excision (with a 1 mm margin) was done in view of the long-standing ulcerated lesion with a history of frequent bleeding and histopathology examination was done in view of the surgically excised lesion. He was reviewed thereafter and no recurrence was noticed at the end of six months.

**Figure 2:**
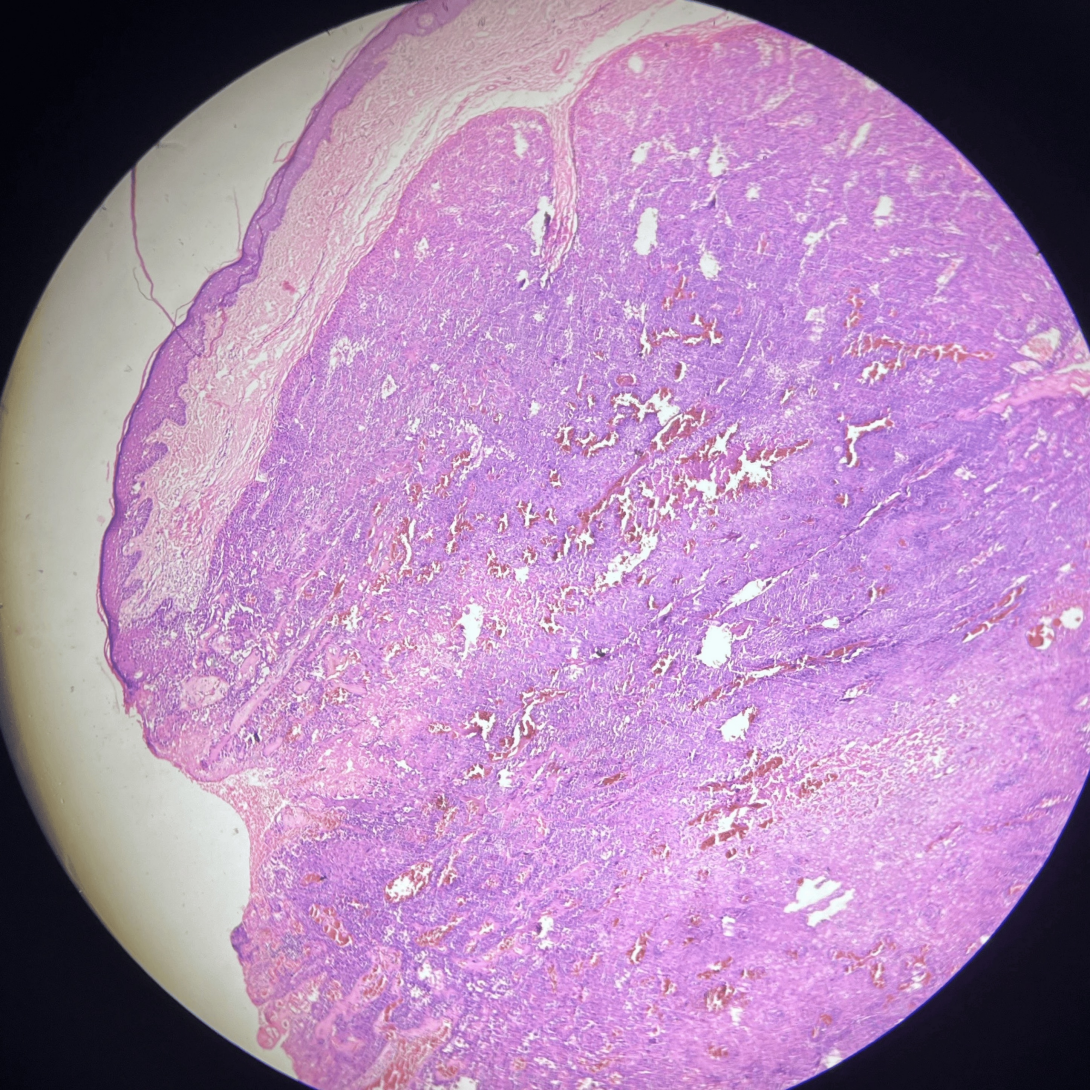
Bulbous proliferation of the follicular infundibulum arising from the overlying epidermis (H & E stain, 4x magnification).

**Figure 3:**
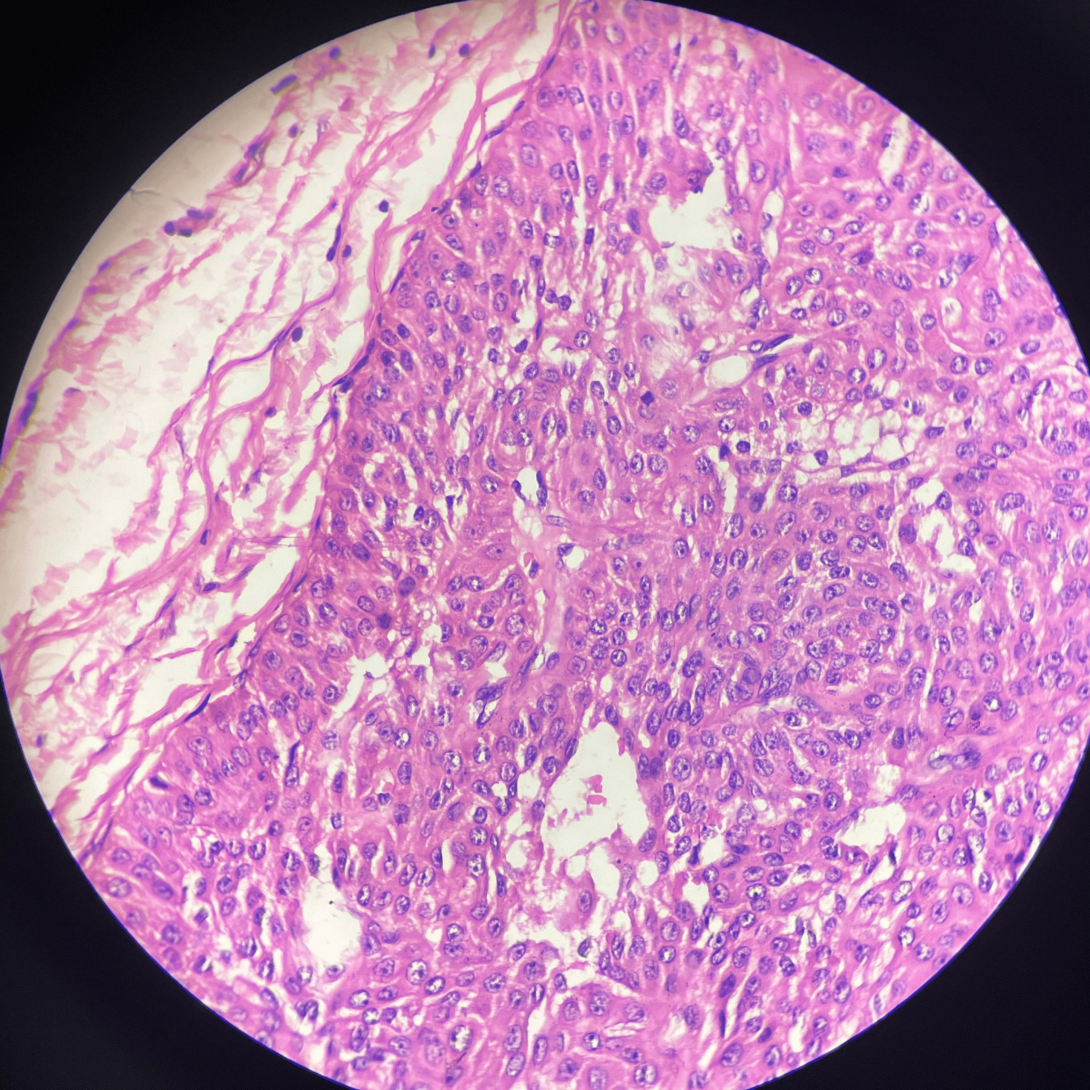
Lobular arrangement of the tumor cells with peripheral palisading and prominent basement membrane (H & E stain, 10x magnification).

**Figure 4:**
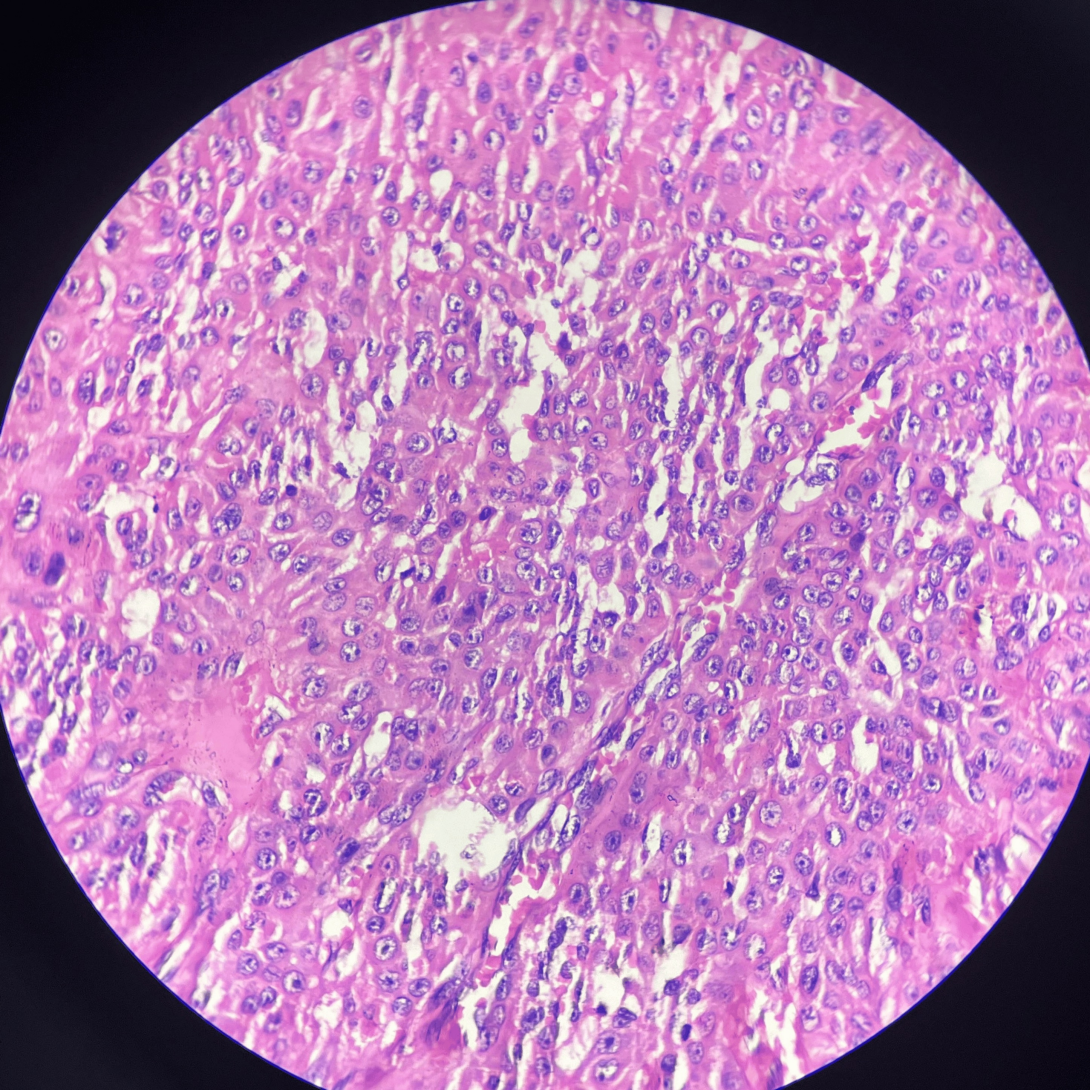
Monomorphic cells with occasional cells showing a clear cytoplasm (H & E stain, 40x magnification).

Histopathology of the excised tissue revealed central ulceration while the rest of the epidermis was thinned out with blunting of rete ridges. The basal cell layer was normal. There was downward proliferation of tumor cells from the overlying epidermis which were arranged in lobules (Figure 2), There was peripheral palisading of cuboidal and columnar cells and focal clear cells resting on the basement membrane. Intervening congested blood vessels were also seen (Figures 3, 4). There was no evidence of dysplasia or malignancy. The histological features were suggestive of a benign cutaneous adnexal neoplasm favoring a trichilemmoma. Thus, the apparently visible PG on clinical examination turned out to be a trichilemmoma.

## Discussion

Appendageal neoplasms of the skin are uncommon. Most of them are benign but show ulceration or necrosis and thus warrant complete surgical excision in view of the possibility of malignancy [4]. Many times, they are confused with other tumors of vascular etiology like PGs, sclerosing hemangioma, etc. PGs often manifest as single, quickly expanding red papules or nodules that are painless most of the time but can bleed spontaneously or with trivial trauma. On the skin, PGs most commonly affect the periungual area of fingers, the feet, lips, the head, the upper trunk, and the perianal area, although it has also been reported on the scalp and usually occurs after trauma [5]. Key features of PGs include numerous vascular spaces lined with endothelium, as well as proliferation of fibroblasts and budding endothelial cells. Additionally, there is often a mixed inflammatory cell infiltration present [6].

Trichilemmomas arise from the outer root sheath of hair follicles [7]. The exact prevalence and incidence of trichilemmomas in the general population are not well-documented, but this disease is considered rare. These tumors are most commonly seen in adults aged 30 to 80, with a median age of 59, and they are rarely found in children [8].

Histopathologically, trichilemmomas are identified by their distinct growth pattern, where they form lobular clusters that extend from the outer layer of the skin into the deeper layer (Figure 2). These tumors are characterized by cells that appear pale or clear and are arranged in a palisading manner along the edges of the lobules (Figures 3, 4). Surrounding these clusters is a thickened eosinophilic basement membrane. Additionally, the surface of the skin over the tumor may show thickened, hyperkeratosis and variable papillomatosis [8].

## Conclusions

In our case, we clinically diagnosed the patient to have a PG and planned for surgical excision for its treatment. However, on histopathological examination, it was found to be a trichilemmoma. When a lesser-known or uncommon entity is observed, histopathological investigation is always the gold standard since histopathological-clinical correlation gives the final diagnosis. The diagnosis of rare diseases requires more than just clinical findings; a high level of clinical suspicion and awareness is needed to further investigate for timely diagnosis. It is necessary for dermatologists to be aware of the various disorders that can be mimicked by PGs.

## References

[1] Headington JT, French J (1962). Primary neoplasms of the hair follicle. Histogenesis and classification. Arch Dermatol.

[2] Ng DW (2016). Trichilemmoma in childhood. J Pediatr Health Care.

[3] Giblin AV, Clover AJ, Athanassopoulos A, Budny PG (2007). Pyogenic granuloma - the quest for optimum treatment: audit of treatment of 408 cases. J Plast Reconstr Aesthet Surg.

[4] Saha A, Das NK, Gharami RC, Chowdhury SN, Datta PK (2011). A clinico-histopathological study of appendageal skin tumors, affecting head and neck region in patients attending the dermatology opd of a tertiary care centre in eastern India. Indian J Dermatol.

[5] Chandra BS, Rao PN (2013). Two cases of giant pyogenic granuloma of scalp. Indian Dermatol Online J.

[6] Marla V, Shrestha A, Goel K, Shrestha S (2016). The histopathological spectrum of pyogenic granuloma: a case series. Case Rep Dent.

[7] Chan P, White SW, Pierson DL, Rodman OG (1979). Trichilemmoma. J Dermatol Surg Oncol.

[8] Dykes RE, Stepien A (2024). Trichilemmoma. StatPearls [Internet].

